# Comparison of the Reductions in LDL-C and Non-HDL-C Induced by the Red Yeast Rice Extract Xuezhikang Between Fasting and Non-fasting States in Patients With Coronary Heart Disease

**DOI:** 10.3389/fcvm.2021.674446

**Published:** 2021-08-09

**Authors:** Li-Yuan Zhu, Xing-Yu Wen, Qun-Yan Xiang, Li-Ling Guo, Jin Xu, Shui-Ping Zhao, Ling Liu

**Affiliations:** ^1^Department of Cardiovascular Medicine, The Second Xiangya Hospital, Central South University, Changsha, China; ^2^Research Institute of Blood Lipid and Atherosclerosis, The Second Xiangya Hospital, Central South University, Changsha, China; ^3^Modern Cardiovascular Disease Clinical Technology Research Center of Hunan Province, Changsha, China; ^4^Cardiovascular Disease Research Center of Hunan Province, Changsha, China; ^5^Xiangya School of Medicine, Central South University, Changsha, China

**Keywords:** non-fasting, coronary heart disease, LDL-C, non-HDL-C, Xuezhikang

## Abstract

**Background:** Xuezhikang, an extract of red yeast rice, effectively lowers fasting blood lipid levels. However, the influence of Xuezhikang on the non-fasting levels of low-density lipoprotein cholesterol (LDL-C) and non-high-density lipoprotein cholesterol (non-HDL-C) has not been explored in Chinese patients with coronary heart disease (CHD).

**Methods:** Fifty CHD patients were enrolled and randomly divided into two groups (*n* = 25 each) to receive 1,200 mg/d of Xuezhikang or a placebo for 6 weeks as routine therapy. Blood lipids were repeatedly measured before and after 6 weeks of treatment at 0, 2, 4, and 6 h after a standard breakfast containing 800 kcal and 50 g of fat.

**Results:** The serum LDL-C levels significantly decreased, from a fasting level of 3.88 mmol/L to non-fasting levels of 2.99, 2.83, and 3.23 mmol/L at 2, 4, and 6 h, respectively, after breakfast (*P* < 0.05). The serum non-HDL-C level mildly increased from a fasting level of 4.29 mmol/L to non-fasting levels of 4.32, 4.38, and 4.34 mmol/L at 2, 4, and 6 h post-prandially, respectively, and the difference reached statistical significance only at 4 and 6 h after breakfast (*P* < 0.05). After 6 weeks of Xuezhikang treatment, the patients had significantly lower fasting and non-fasting serum levels of LDL-C and non-HDL-C (*P* < 0.05) than at pretreatment. The LDL-C levels were reduced by 27.8, 28.1, 26.2, and 25.3% at 0, 2, 4, and 6 h, respectively, and the non-HDL-C levels were reduced by 27.6, 28.7, 29.0, and 28.0% at 0, 2, 4, and 6 h, respectively, after breakfast. No significant difference was found in the percent reductions in the LDL-C and non-HDL-C levels among the four different time-points.

**Conclusions:** Six weeks of Xuezhikang treatment significantly decreased LDL-C and non-HDL-C levels, with similar percent reductions in fasting and non-fasting states in CHD patients, indicating that the percent change in non-fasting LDL-C or non-HDL-C could replace that in the fasting state for evaluation the efficacy of cholesterol control in CHD patients who are unwilling or unable to fast.

## Introduction

According to the Chinese Cardiovascular Report 2019, 11 million Chinese individuals suffered from coronary heart disease (CHD). The mortality rate of CHD was 115.32/100,000 in urban residents and 122.04/100,000 in rural residents (1). Compared with fasting hypertriglyceridemia, elevated fasting low-density lipoprotein cholesterol (LDL-C) levels are more closely associated with CHD, although evidence also shows that non-fasting hypertriglyceridemia is an independent risk factor for CHD (2). Hypertriglyceridemia represents an increase in triglyceride (TG)-rich lipoproteins and their remnant-like particles (RLPs) in peripheral circulation. Remnant-like particles are considered to be as atherosclerotic as low-density lipoprotein (LDL). Reducing LDL-C levels is the primary goal of cholesterol control, while the secondary goal is to control the level of non-high-density lipoprotein cholesterol (non-HDL-C) (3).

Atherogenesis is a non-fasting phenomenon (4). Non-fasting lipids are initially detected after a high-fat meal, and more attention has been given to TG than to LDL-C or non-HDL-C (5, 6). Recently, non-fasting blood lipids after a daily meal were reported in the prospective studies with large populations (7). Additionally, it was recommended that non-fasting blood samples should be routinely used for the assessment of lipid profiles in CHD patients on stable drug therapy or those who prefer non-fasting lipid detection (8, 9). However, it remains unclear whether the detection of non-fasting blood lipids is appropriate during follow-up in CHD patients receiving their first treatment with statins.

After a daily meal, the levels of certain non-fasting cholesterol parameters are reduced (10). The maximal mean reduction in the LDL-C or non-HDL-C level at 1–6 h after habitual food intake was only 0.2 mmol/L, which was considered non-significant in a population from Copenhagen (11). However, Chinese subjects seemed to have a larger drop in non-fasting LDL-C levels after a daily breakfast; the decrease was more than 0.3 mmol/L, when the LDL-C level was calculated according to the Friedewald formula (12). The non-fasting reduction in LDL-C may influence the evaluation of cholesterol control. Thus, it could be more appropriate to evaluate the efficacy of cholesterol control according to the percent reduction in LDL-C rather than the absolute LDL-C level in the non-fasting state.

Patients with type 2 diabetes, functional dyspepsia and CHD could benefit from dietary supplements, including red yeast rice. As a natural statin, Xuezhikang, which is extracted from red yeast rice, has been recommended for the secondary prevention of CHD by the Guidelines for the Prevention and Treatment of Dyslipidemia in Chinese Adults (13, 14). Xuezhikang significantly improved the prognosis of Chinese patients with CHD by comprehensively regulating lipids, including by reducing of non-fasting TG and lipoprotein(a) levels (15, 16), although its effect on non-fasting LDL-C and non-HDL-C levels was never mentioned. The high-fat breakfast with 800 kcal that was used in our previous studies (16–20) had relatively fewer calories than those used in other studies (21, 22), and this calorie level could be close to that of the habitual or daily breakfasts of some individuals. Thus, we aimed to explore the effects of short-term Xuezhikang treatment (1,200 mg/d) on the non-fasting LDL-C and non-HDL-C levels of CHD patients who were accustomed to consuming a standard breakfast with 800 kcal and 50 g of fat, and to compare the percent reduction in the LDL-C and non-HDL-C levels between the fasting and non-fasting states.

## Materials and Methods

### Study Design and Population

The study protocol was approved by the Ethics Committee of Central South University (Hunan, China) and conformed to the ethical guidelines of the 1975 Declaration of Helsinki. Written informed consent was provided by all the participants. This randomized, single-blind, placebo-controlled study was performed in consecutive patients (*n* = 50). Patients who visited the second Xiangya Hospital between February 2001 and January 2002 for diagnostic evaluation or treatment were recruited. Enrolled patients were divided into the Xuezhikang and placebo groups at a 1:1 ratio according to an odd or even number that they randomly selected by themselves. Their dietary habits, nutrient intake, quantity of food intake, and daily activity were investigated by an open-question interview using a nutrition and health questionnaire (23, 24).

The inclusion criteria were as follows: men or women aged ≥18 years; New York Heart Association (NYHA) class I or II; CHD that was defined as a history of myocardial infarction and/or angiographically proven coronary artery stenosis ≥50% with angina pectoris; subjects who tolerated a breakfast containing at least 800 kcal and 50 g of fat well.

The exclusion criteria were as follows: diabetes; thyroid diseases; liver and kidney diseases; malignancy; chronic consuming diseases, including malignant tumors, tuberculosis, chronic atrophic gastritis, severe trauma, burns, systemic lupus erythematosus, chronic suppurative infection, and chronic blood loss; and the use of oral hypoglycemic or hypolipidemic agents.

All the patients had a 4-week dietary advisory period. After at least 12 h of overnight fasting, the patients were given a standard breakfast with 800 kcal and 50 g of fat. Next, they either received Xuezhikang (1,200 mg/d, 600 mg of cholestin per capsule, WBL Peking University Biotech Co., Ltd., China) or the placebo. After 6 weeks, the same standard breakfast was repeated. All the patients maintained a steady diet according to lipid-lowering dietary advice and accepted routine therapy, including aspirin, metoprolol, fosinopril, and nitrates, during a 6-week follow-up. No patients dropped out of the study during the 6-week follow-up.

### Standard Breakfast and Collection of Blood Samples

The standard breakfast in this study contained 800 kcal, 50 g of fat, 28 g of protein, and 60 g of carbohydrates. Blood samples were taken before and at 2, 4, and 6 h after the meal. During the 6-h test, the patients were not allowed to smoke, drink wine, or eat any foods, with the option of consuming a little water. Vigorous exercise, including running and talking loudly, was forbidden, and only slow walking was allowed in a certain range of ward areas. Intravenous infusion was prohibited until the last blood sample was collected.

### Lipid Profile Measurements

Blood samples were separated at 4°C and stored at −20°C. The serum levels of total cholesterol (TC), TG, and high-density lipoprotein cholesterol (HDL-C) were measured using an automatic biochemistry analyzer (Hitachi 7170, Tokyo, Japan) by a specialist who was blinded to this study. The LDL-C levels were calculated according to the Friedewald formula, i.e., LDL-C = TC – HDL-C – TG/2.2 (mmol/L), when the TG level was <4.5 mmol/L; otherwise, it was measured by using a commercial direct method (Daiichi Pure Chemicals Co., Ltd. Tokyo, Japan). The cholesterol content in RLPs is termed remnant lipoprotein cholesterol (RC). The non-HDL-C and RC levels were estimated according to two formulas, non-HDL-C = TC – HDL-C and RC = non-HDL-C – LDL-C, respectively.

### Statistical Analysis

The data were analyzed by SPSS version 23.0 (IBM Corp, Armonk, NY, USA) and GraphPad Prism version 8.0.2 software (GraphPad Corp, San Diego, CA, USA). Quantitative variables are expressed as the mean ± SD, mean ± SEM or median and interquartile, and categorical variables are expressed as numbers or percentages (%). Intragroup comparisons among multiple time points were performed by one-way ANOVA for quantitative variables, and comparisons between two different time points were performed by *t*-test. When quantitative variable did not meet the normality assumption of ANOVA or Wilcoxon test for *post-hoc* analysis, the Kruskal-Wallis test was performed. The difference in intergroup means was analyzed by independent *t*-test. Categorical variables were compared using χ^2^ analysis. The total area under the curve (tAUC) of each lipid parameter was estimated by using linear trapezoidal methods after breakfast as before (20). Statistical significance was assumed at a two-tailed value of *P* < 0.05.

## Results

### Clinical Characteristics of the Recruited Patients

The baseline characteristics, including sex, age, body mass index (BMI), heart rate, smoking habits, hypertension status, blood pressure, fasting blood glucose, and creatinine, were roughly matched in the Xuezhikang (XZK) and control (CON) groups. Moreover, no significant difference was found in the fasting levels of TG, TC, LDL-C, HDL-C, non-HDL-C, or RC between the two groups (Table 1).

**Table 1:** Clinical characteristics of the patients.

	**XZK group (*n* = 25)**	**CON group (*n* = 25)**	***P*-value**
Age (years)	57.88 ± 5.69	58.64 ± 5.67	0.639
Male (*n*)	16	16	1.000
BMI (kg/m^2^)	24.79 ± 2.16	24.91 ± 1.15	0.815
Smoker (*n*)	8	8	1.000
Hypertension (*n*)	12	12	1.000
SBP (mmHg)	125 ± 16	129 ± 22	0.526
DBP (mmHg)	81 ± 10	79 ± 7	0.432
Heart rate (1/min)	77 ± 7	77 ± 6	0.964
FBS (mmol/L)	5.46 ± 0.53	5.35 ± 0.44	0.437
Creatinine (μmmol/L)	108.17 ± 9.75	110.16 ± 7.37	0.421
TG (mmol/L)	2.00 ± 0.52	1.95 ± 0.34	0.686
TC (mmol/L)	5.47 ± 0.55	5.53 ± 0.40	0.628
LDL-C (mmol/L)	3.34 ± 0.41	3.33 ± 0.33	0.973
HDL-C (mmol/L)	1.15 ± 0.19	1.16 ± 0.13	0.767
Non-HDL-C(mmol/L)	4.32 ± 0.54	4.37 ± 0.35	0.685
RC (mmol/L)	0.98 ± 0.56	1.04 ± 0.36	0.672

*BMI, body mass index; SBP, systolic blood pressure; DBP, diastolic blood pressure; FBS, fasting blood glucose; TG, triglyceride; TC, total plasma cholesterol; LDL-C, low-density lipoprotein cholesterol; HDL-C, high-density lipoprotein cholesterol; RC, remnant lipoprotein cholesterol. The data are expressed as the mean ± SD or numbers*.

### Effects of the 800 kcal Breakfast on Blood Lipids

**Figure 1:**
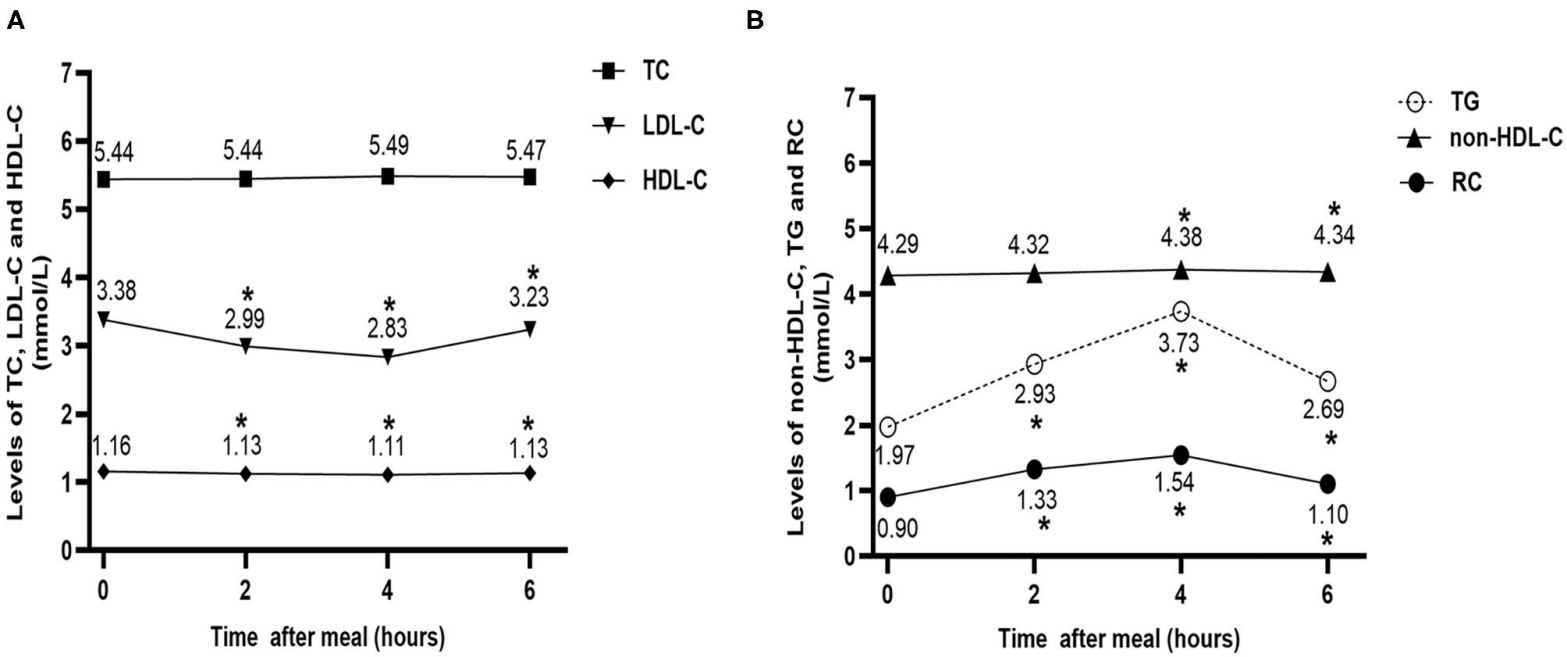
Effect of a high-fat meal on non-fasting levels of blood lipids in all patients at baseline (*n* = 50). **(A)** Changes in non-fasting serum levels of TC, LDL-C, and HDL-C after a high-fat meal at baseline. **(B)** Changes in non-fasting serum levels of TG, non-HDL-C, and RC after a high-fat meal at baseline. *P < 0.05 when compared with the fasting level of the same parameter. Data are expressed as the mean ± SEM. TC, total cholesterol (A); LDL-C, low-density lipoprotein cholesterol (B); HDL-C, high-density lipoprotein cholesterol (C); non-HDL-C, non-high-density lipoprotein cholesterol (D); TG, triglyceride (E); RC, remnant cholesterol (F).

To analyze the changes in the non-fasting blood lipids in CHD patients at baseline, we pooled all the patients together (*n* = 50). No significant difference was found in in TC levels between the fasting and non-fasting states. The other two cholesterol parameters decreased significantly after breakfast (*P* < 0.05); although the reduction in non-fasting HDL-C was very mild, that in LDL-C was relatively obvious, especially 4 h post-prandially. Additionally, the non-fasting non-HDL-C, TG, and RC levels increased significantly after a high-fat breakfast (*P* < 0.05). However, the elevation of non-fasting non-HDL-C was very mild, while that of TG and RC was higher, particularly 4 h post-prandially (Figures 1A,B).

### Changes in the Non-fasting Levels of Blood Lipids Between the Two Groups Before and After Six Weeks

**Figure 2:**
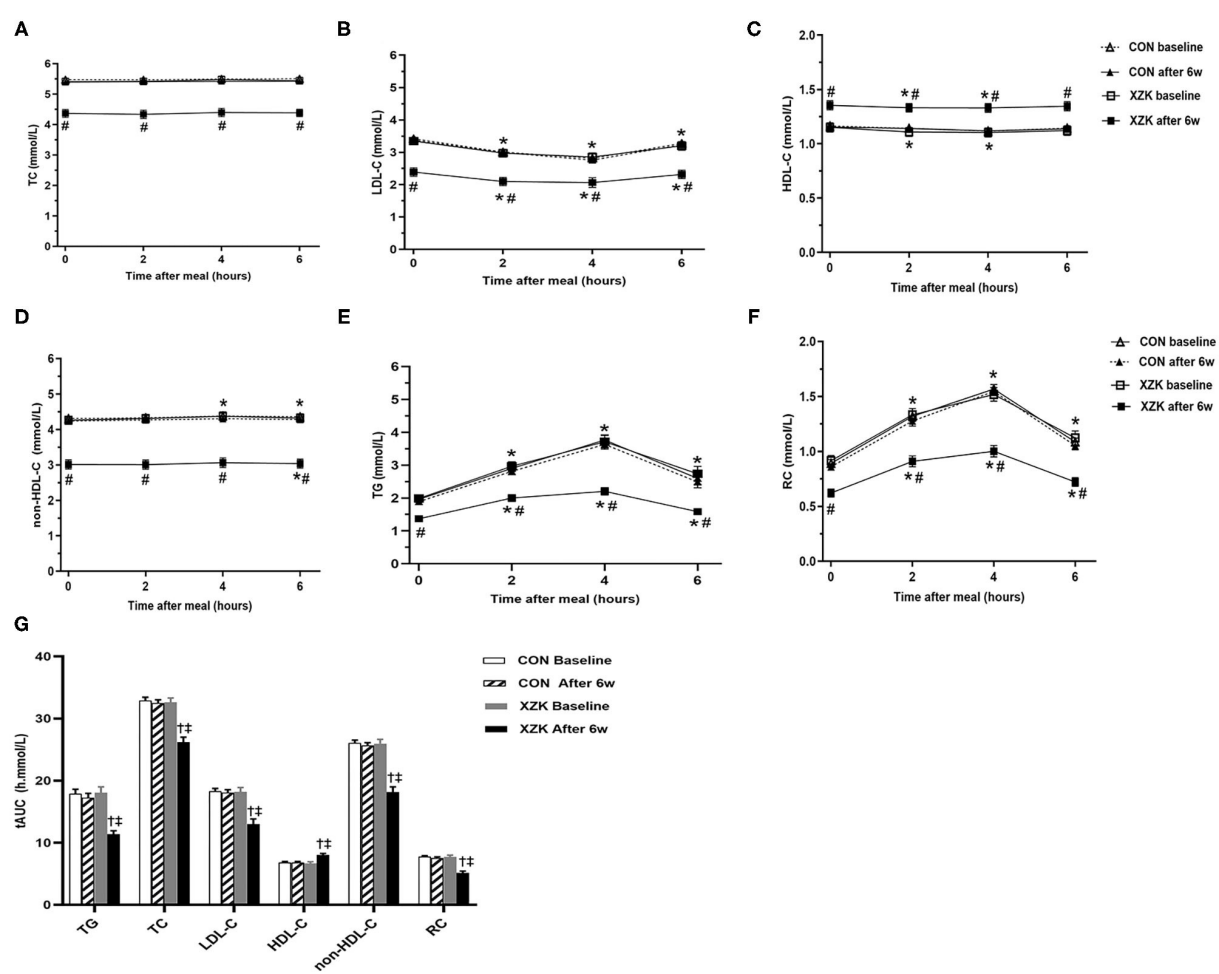
Effect of XZK treatment on non-fasting levels of blood lipids after a high-fat meal. **(A–F)** Changes in fasting and non-fasting levels of blood lipids between the two groups before and after 6 w. *P < 0.05 when compared with the fasting level in the XZK group. #*P* < 0.05 when compared with the CON group at the same time point after 6 weeks. Data are expressed as the mean ± SEM. **(G)** Comparisons of the tAUC for blood lipids after a high-fat meal before and after 6 weeks in the two groups. ^†^*P* < 0.001 when compared with the CON group after 6 weeks. ^‡^*P* < 0.001 when compared with the XZK group at baseline. Data are expressed as the mean ± SEM.

The XZK and CON groups showed similar changes in non-fasting blood lipids after a high-fat breakfast compared to baseline. Six weeks of Xuezhikang treatment significantly increased the HDL-C levels and decreased the TG, TC, LDL-C, non-HDL-C, and RC levels in both the fasting and non-fasting states (*P* < 0.05). However, no significant difference was found in the fasting levels of blood lipids and their non-fasting changes before and after 6 weeks in the CON group (Figures 2A–F).

Next, the tAUC was calculated to reflect overall changes in the non-fasting blood lipids within 6 h after a high-fat breakfast. No significant difference was found in the baseline tAUC for TG (18.0 vs. 17.9 h·mmol/L), TC (32.5 vs. 32.9 h·mmol/L), LDL-C (19.1 vs. 19.2 h·mmol/L), HDL-C (6.7 vs. 6.8 h·mmol/L), non-HDL-C (25.7 vs. 26.1 h·mmol/L), or RC (6.7 vs. 6.9 h·mmol/L) between the groups. After 6 weeks, the tAUC values for TG, TC, LDL-C, non-HDL-C, and RC were significantly lower, while that for HDL-C was significantly higher, than the baseline values in the XZK group (*P* < 0.001) but not in the CON group (Figure 2G).

### Comparisons of the Fasting and Non-fasting Percent Reductions in the LDL-C and Non-HDL-C Levels Before and After Six Weeks in the XZK Group

After 6 weeks of Xuezhikang treatment, the serum LDL-C level showed a reduction of 27.8% in the fasting state and 28.1, 26.2, and 25.3% at 2, 4, and 6 h post-prandially, respectively. The serum non-HDL-C levels showed a reduction of 27.6% in the fasting state and 28.7, 29.0, and 28.0% at 2, 4, and 6 h post-prandially, respectively. No significant difference was found in the percent reductions in the LDL-C and non-HDL-C levels between the fasting and non-fasting time points (Table 2).

**Table 2:** Comparisons of the percent reductions in the LDL-C and non-HDL-C levels among different time points after six weeks of Xuezhikang treatment (*n* = 25).

	**0 h**	**2 h**	**4 h**	**6 h**	**H**	***P-*value**
LDL-C	27.8%	28.1%	28.1%	25.3%	0.222	0.974
	(21.2–33.8)	(16.4–38.0)	(16.4–38.0)	(16.7–37.0)		
non-HDL-C	27.6%	28.7%	29.0%	28.0%	0.068	0.995
	(22.0–33.9)	(22.9–35.5)	(23.7–36.0)	(22.2–33.3)		

*The data are expressed as the median and interquartile range*.

## Discussion

In this study, the patients consumed an identical breakfast before and after 6 weeks of Xuezhikang treatment, and non-fasting blood lipids were detected at the same time-points after breakfast. It was found that the percent reduction in LDL-C at any time point after breakfast was similar to that in the fasting state, suggesting that the percent reduction in non-fasting LDL-C level can be used to evaluate the efficacy of cholesterol control for CHD patients who are unwilling or unable to keeping the fast state, as can the non-fasting non-HDL-C level. This means that non-fasting LDL-C and non-HDL-C levels can be used in place of their fasting values when evaluating the efficacy of statin treatment in specific CHD patients.

Among all the cholesterol parameters, the LDL-C levels were obviously reduced after breakfast. The TC levels were the most stable after breakfast, followed by the HDL-C and non-HDL-C levels, the changes did not exceed 0.1 mmol/L in this study. However, the RC level increased with increasing TG levels. Some scholars speculated the reduced in non-fasting LDL-C levels were likely induced by hemodilution due to fluid intake (11, 25). Others attributed the decrease to biological rhythm and found that LDL-C and HDL-C levels were significantly lower from after breakfast until midnight than they were before breakfast (26). Although the potential cause of the reduction in the non-fasting LDL-C levels in this study was not very clear, we should cautiously evaluate the cholesterol-lowering effect during follow-up if the LDL-C level was detected in a non-fasting state. For example, if the fasting LDL-C goal was <1.8 mmol/L, it was difficult to decide whether a non-fasting LDL-C level of 1.65 mmol/L reached the target. Thus, evaluating the non-fasting LDL-C level according to the fasting LDL-C goal may be inappropriate.

With the update of the Chinese and Western guidelines for the management of dyslipidemia in adults, evaluation of the percent reduction in LDL-C level has become increasingly important (13, 27, 28). For a non-negligible reduction in LDL-C levels after breakfast in this study, the percent reduction was calculated to compare the effects of Xuezhikang on the LDL-C levels between the fasting level and the levels at the non-fasting time points. After 6 weeks, LDL-C levels presented similar changes at four different time points, with a percent reduction between 25.3 and 28.2%. More importantly, no significant difference was found in the percent reductions in LDL-C level among the four different time points. Thus, if the LDL-C level was detected after a habitual breakfast in the first visit in one patient, it can be detected repeatedly after an identical breakfast at the second visit 6 weeks later. That is, non-fasting LDL-C levels could even be used to evaluate cholesterol control in unstable patients. Considering those the CHD patients who regularly consumed a daily breakfast with ≥800 kcal and ≥50 g of fat, the standard breakfast in this study can be regarded as a customary breakfast. Thus, the findings from this study could apply to CHD patients with similar dietary habits.

In the present study, the percent reduction in the LDL-C level was <27.8%, which was very close to that reported by other studies, including 28.5% by the China Coronary Secondary Prevention Study (CCSPS) and 27% by a Food and Drug Administration Phase II clinical study (29). With every 1% decrease in the LDL-C level, the relative risk of cardiovascular events is reduced by 1% (30). Because Chinese patients with CHD had lower baseline LDL-C levels than Western patients (1, 31, 32), a nearly 30% reduction in LDL-C induced by a 6-week Xuezhikang treatment lowered LDL-C level to <2.6 mmol/L in 76% of CHD patients, representing LDL-C goal recommended by the guidelines of the National Cholesterol Education Program in 2002 (33). Considering that this study was completed in 2002, a nearly 30% reduction in LDL-C level was clinically significant at that time.

Compared with the LDL-C levels, the non-HDL-C levels changed slightly after breakfast in this study, which is likely related to the obvious increase in RC levels. The non-HDL-C level includes the LDL-C level and the RC level at each time-point. The percent reduction in the non-HDL-C level was slightly greater than that in the LDL-C level at each non-fasting time point, likely because Xuezhikang inhibited the increase in the non-fasting TG and RC levels. Similar to the LDL-C level, the non-HDL-C level also showed very similar percent reductions among the four different time points. This finding indicates that non-HDL-C levels can be detected and evaluated in the non-fasting state during follow-up for those who are unwilling or unable to maintain the fasting state (27, 34, 35).

The mechanism by which Xuezhikang reduces blood lipids is complex. Xuezhikang contains natural lovastatin (i.e., monacolin K) and 12 other natural monacolins (24 mg in each Xuezhikang capsule) that are statin homologs. Additionally, it comprises other ingredients, such as alkaloids, flavonoids, ergosterol and unsaturated fatty acids (36). Compared with 10 mg of lovastatin, 1,200 mg of Xuezhikang showed more potent effects in lowering cholesterol and the TG levels (37). China Coronary Secondary Prevention Study demonstrated that Xuezhikang significantly decreased the risk of cardiovascular events and total mortality by 30 and 33%, respectively, in Chinese patients with CHD, a finding that was not completely explained by the decrease in LDL-C induced by natural statins in Xuezhikang (29). Other components of Xuezhikang, such as unsaturated fatty acids, sterols, and flavonoids, may also play essential roles in cardiovascular protection (36). New evidence has shown that unsaturated fatty acids not only have antioxidative and TG-lowering effects but also further reduce cardiovascular events based on statin treatment (38–40).

The safety of Xuezhikang was a concern (41). However, according to a recent meta-analysis of 53 randomized controlled trials with a total of 8,535 patients (4,437 in the red yeast rice treatment arm and 4,303 in the control arm), the use of monacolin K is not associated with an increased risk of muscular adverse events (OR 0.94, 95% CI, 0.53–1.65) (42). In the real world, some patients could take Xuezhikang because of their intolerance to synthetic statins (43). Xuezhikang should be taken as two capsules orally after meals twice a day (44). Dual lipid-lowering therapy with statins and a non-statin agent has promoted coronary atherosclerosis regression and improved cardiovascular outcomes in CHD patients. Pharmacological inhibition of cholesterol absorption (with ezetimibe) and PCSK9 activity (with evolocumab or alirocumab) may show a better therapeutic effect on LDL-C metabolism in statin-treated patients (45).

This study has several limitations. First, this study was a single-blind clinical observational study with a small size. According to the previous recommendations of international guidelines around 2000 (33), statin treatment was initiated after ineffective lifestyle interventions in CHD patients, explaining why approximately half of the participants in this study did not receive Xuezhikang treatment immediately after the CHD diagnosis. A randomized, double-blind clinical trial with a larger sample is warranted in the future. Second, more relevant lipid profiles and inflammatory markers should be detected during throughout the day in the future, although a certain type of breakfast may trigger the fluctuation of inflammation and metabolic parameters that are linked to biological rhythms. Third, the Women's Health Study showed that directly measured RC and small LDL-C are related to multiple incident cardiovascular outcomes (46). The directly measured RC level should give more attention in our future work since it is derived by calculation only in this study.

## Conclusion

Six-week of Xuezhikang treatment significantly decreased LDL-C and non-HDL-C levels, with similar percent reductions in fasting and non-fasting states in CHD patients, indicating that the percent change in non-fasting LDL-C or non-HDL-C could replace that in the fasting one to evaluate the efficacy of cholesterol control in CHD patients who are unwilling or unable to fast.

## Data Availability Statement

The raw data supporting the conclusions of this article will be made available by the authors, without undue reservation.

## Ethics Statement

The studies involving human participants were reviewed and approved by the Ethics Committee of the Second Xiangya Hospital of Central South University. The patients/participants provided their written informed consent to participate in this study.

## Author Contributions

L-YZ and LL wrote the manuscript and reviewed the literature. LL and S-PZ designed the study and collected the data. L-YZ, X-YW, Q-YX, L-LG, and JX analyzed the data and prepared the figures and tables. All authors approved the final manuscript.

## Conflict of Interest

The authors declare that the research was conducted in the absence of any commercial or financial relationships that could be construed as a potential conflict of interest.

## Publisher's Note

All claims expressed in this article are solely those of the authors and do not necessarily represent those of their affiliated organizations, or those of the publisher, the editors and the reviewers. Any product that may be evaluated in this article, or claim that may be made by its manufacturer, is not guaranteed or endorsed by the publisher.

## Abbreviations

CHD: coronary heart disease
TC: total cholesterol
LDL-C: low-density lipoprotein cholesterol
HDL-C: high-density lipoprotein cholesterol
non-HDL-C: non-high-density lipoprotein cholesterol
TG: triglyceride
RLP: remnant-like particle
RC: remnant lipoprotein cholesterol
BMI: body mass index
SBP: systolic blood pressure
DBP: diastolic blood pressure
FBS: fasting blood sugar
CCSPS: China Coronary Secondary Prevention Study.
